# Loss of Tuberous Sclerosis Complex 2 confers inflammation via dysregulation of Nuclear factor kappa-light-chain-enhancer of activated B cells

**DOI:** 10.21203/rs.3.rs-4569999/v1

**Published:** 2024-07-15

**Authors:** Darius K. McPhail, Mohammad A.M. Alzahrani, Katie R. Martin, Brian L. Calver, Adrian J. Harwood, Jeffrey P. MacKeigan, David M. Davies, Andrew R. Tee

**Affiliations:** Cardiff University; Cardiff University; Michigan State University; Cardiff University; Cardiff University; Michigan State University; South West Wales Cancer Centre, Singleton Hospital; Cardiff University

**Keywords:** TSC, mTOR, NF-κB, STAT3, IL-6, rapamycin, inflammation

## Abstract

**Background:**

Aberrant activation of mTORC1 is clearly defined in TSC, causing uncontrolled cell growth. While mTORC1 inhibitors show efficacy to stabilise tumour growth in TSC, they are not fully curative. Disease facets of TSC that are not restored with mTOR inhibitors might involve NF-κB. The study aimed to characterise NF-κB in the context of TSC.

**Results:**

Enrichment of NF-κB-regulated genes was observed in TSC patient tumours, SEN/SEGAs, cortical tubers and a TSC tumour-derived cell line (621 – 101). Highlighting an inflammatory component of TSC, TSC cell models showed an elevated level of NF-κB and STAT3 activation. Herein, we report a dysregulated inflammatory phenotype of *TSC2*-deficient cells where NF-κB promotes autocrine signalling involving IL-6. Of importance, mTORC1 inhibition does not block this inflammatory signal to promote STAT3, while NF-κB inhibition was much more effective. Combined mTORC1 and NF-κB inhibition was potent at preventing anchorage-independent growth of *TSC2*-deficient cells, and unlike mTORC1 inhibition alone was sufficient to prevent colony regrowth after cessation of treatment.

**Conclusion:**

This study reveals autocrine signalling crosstalk between NF-κB and STAT3 in TSC cell models. Furthermore, the data presented indicate that NF-κB pathway inhibitors could be a viable adjunct therapy with the current mTOR inhibitors to treat TSC.

## Background

Tuberous Sclerosis Complex (TSC) is a rare, autosomal dominant genetic condition caused by inactivating mutations in either the *TSC1* or *TSC2* genes. TSC patients are predisposed to kidney, skin, brain, and heart tumours (reviewed in [1]). Renal angiomyolipomas (AML) are highly vascularised benign tumours containing both smooth muscle and adipose tissue occurring in ~ 80% of adult TSC patients and are the primary cause of mortality past the age of 30 [2]. TSC-associated brain lesions include subependymal nodules (SEN) and subependymal giant cell astrocytomas (SEGA) that can result in hydrocephalus [3]. Additionally, TSC patients often present with cortical tubers, which are believed to be the epileptic foci in the majority of TSC cases [4]. White matter abnormalities are also common in TSC patients (up to 95%) and likely contribute to the onset, frequency, and severity of seizures [5]. Approximately 90% of TSC patients will experience a seizure within their lifetime. Seizures can be refractive to standard anti-epileptic medications, making seizures difficult to treat in approximately one-third of TSC patients [6]. Furthermore, 50% of TSC patients will have some degree of intellectual disability [7].

Currently, one key feature of TSC1/TSC2 biology is well understood: the ability of the TSC1/2 tumour suppressor complex to inhibit growth signalling through mechanistic target of rapamycin complex 1 (mTORC1). The small G protein Rheb, which directly activates mTORC1 kinase activity when GTP-bound, is negatively regulated by the GTPase activating protein (GAP) domain of TSC2 [8]. Consequently, inactivating mutations within either TSC1 or TSC2 favours GTP-loading of Rheb and aberrant protein kinase activity of mTORC1, leading to uncontrolled cell growth. mTORC1 inhibitors are now used worldwide to treat TSC patients to stabilise disease. Long-term treatments with mTORC1 inhibitors (> 3 years) in TSC patients was found to markedly improve seizures that were refractory to conventional antiepileptic drugs [9]. Tumour volumes are also reduced by mTORC1 inhibitors, with both angiomyolipomas and SEGAs being reduced by > 60%. While mTORC1 inhibitors alleviate many disease traits of TSC, they do not restore disease to normal (reviewed in 10]). For instance, tumours do not regress completely and grow back when therapy stops. A greater understanding of how the loss of either *TSC1* or *TSC2* can drive disease is required before more curative therapies can be developed for TSC.

In this study, we examined differentially expressed genes in TSC patient tumours that highlighted gene sets involved in oxidative stress and inflammation. Oxidative stress is known to activate redox-sensitive transcription factors, such as nuclear factor kappa B (NF-κB). NF-κB is involved in the survival, growth, and migration of cancer cells (reviewed in [11]) and is stimulated by a variety of growth factors or cytokines (reviewed in [12]). Briefly, NF-κB subunits, RelA (p65) and RelB are expressed ubiquitously and reside in their inactive forms in the cytoplasm. The RelA and RelB subunits possess transcriptional activation domains. To activate NF-κB, NF-κB inhibitor alpha (commonly referred to as IκBα) is phosphorylated and inactivated by the IκB kinase (IKK) complex. This causes dissociation, ubiquitination, and subsequent degradation of IκBα. IKK also phosphorylates RelA at S536, promoting the transcriptional activity of NF-κB via the association of homo or heterodimers of NF-κB subunits, most commonly RelA/NF-κB1. These activator inputs unmask the nuclear localisation signals within NF-κB subunits, leading to their nuclear translocation and transcription of pro-inflammatory NF-κB genes.

STAT3 is a pro-inflammatory transcription factor that promotes oncogenesis by enhancing tumour survival, motility, and cell proliferation [13]. Phosphorylation of Y705 is the most well-known mechanism of STAT3 activation, and typically occurs downstream of cytokine stimulation. For example, IL-6 stimulation results in phosphorylation of Y705-STAT3. This leads to STAT3 dimerisation and subsequent translocation to the nucleus, where STAT3 homodimers promote pro-inflammatory gene activation [14]. STAT3 possesses a phosphorylation site on S727, although the functional role of this is poorly understood. S727 phosphorylation is believed to negatively regulate Y705 STAT3 phosphorylation, thus reducing STAT3 inflammatory activity [15]. However, other studies report that S727 phosphorylation is required (alongside Y705 phosphorylation) for maximal STAT3 activation (and tumorigenic signalling) [16].

While mTOR inhibitors demonstrate significant clinical applicability, their effect is often limited and mTOR inhibitors are relatively ineffective at reducing various disease-associated signalling pathways, such as NF-κB, STAT3, and HIF-1α [17]. For this reason, investigation into inflammatory pathways may offer an alternative treatment option for TSC.

Current evidence of NF-κB in TSC is limited and suggests varied dysregulation. One study reported a context-dependent role of TSC2 in NF-κB activity [18]. Small interfering RNA knockdown of TSC2 was found to increase the activity of NF-κB, however this effect was only observed in cells with non-functional PTEN. It was believed that this occurred downstream of mTORC1. Conversely, the same study reported that when PTEN was restored, *TSC2* knockdown resulted in a decrease in NF-κB activation. This highlights the context-dependent role of TSC2 in the regulation of NF-κB. Another study revealed that mTORC1 inhibition impacted NF-κB activation within *TSC2*-deficient immune cells [19]. Notably, in the *TSC2*-deficient cells, the transactivation domain of RelA was inactivated by mTORC1-dependent phosphorylation resulting in reduced NF-κB activity. Inhibition of mTORC1 reversed the reduction of NF-κB activity and resulted in hyperactivation of NF-κB. Given the possible complex role of dysfunctional NF-κB activity in the pathophysiology of TSC, herein we sought to further elucidate NF-κB in the context of TSC.

## Methods

### Cell culture and drug treatments

621 – 101 *TSC2*-deficient (*TSC2*−) cells were derived from the renal AML of a TSC patient and possess a homozygous missense mutation in *TSC2* (G1832A), resulting in an R611Q amino acid substitution [20]. Wild-type human *TSC2* was re-expressed to generate 621 – 103 (*TSC2*+) AML cells [21]. *Tsc2*(−/−) and *Tsc2*(+/+) mouse embryonic fibroblasts (MEF) are immortalised with *Tp53*(−/−), which was originally derived from early-stage embryos from an interbreeding study [22]. Eker rat leiomyoma-derived *Tsc2*-deficient cells (ELT3-V3) and matched controls re-expressing *Tsc2* (ELT3-T3) were generated by Astrinidis *et al*. [23]. and was gifted by C. Walker (M.D. Anderson Cancer Center, Houston, USA). Cell lines were maintained at 37°C, 5% CO_2_ in a humidified incubator. When indicated, cells were incubated under hypoxic conditions and 1% O_2_ was achieved with N_2_ displacement. Cells were cultured in DMEM (Gibco^™^, Thermo Fisher Scientific, Oxford, UK) on Techno Plastic Products^™^ coated tissue culture plasticware (Helena Biosciences Europe, Gateshead, UK), supplemented with fetal bovine serum (FBS) at either 10% (*v/v*) or 15% (*v/v*) for MEF and ELT3 cells or AML cells, respectively, with 50 IU/mL penicillin and streptomycin. 100 μM rapamycin and 20 mM C188–9 (Merck Life Science UK Ltd, Gillingham, UK) and 20 mM BMS-345541 (Selleck Chemicals GmBh, Munich, Germany) drug stocks were made up in dimethylsulfoxide (DMSO) and stored as single use aliquots at −80 °C. Drugs were added to the culture media at a consistent % (*v/v*) DMSO per condition, without exceeding 0.5% (*v/v*) DMSO. Tumour necrosis factor α (TNFα) and interleukin 6 (IL-6) (purchased from Abcam, Cambridge, UK) were resuspended in ddH_2_O containing 0.2% (*w/v*) bovin serum albumin (BSA) to 100 μg/mL and 50 μg/mL, respectively (stored as single use aliquots at −80 °C). Cell passage was kept < 30 in AMLs and ELT3, and < 45 in MEFs. Mycoplasma-free frozen cell stocks were used; all cells were routinely checked with Venor GeM advance mycoplasma detection kit (Minerva Biolabs, Berlin, Germany) as per manufacturers guidelines and were negative to the presence of mycoplasma spp.

### Western blotting

Cells were seeded on 60 mm plates and grown to 70–80% confluency prior to treatments. To generate nuclear cell lysates, cells were washed in ice-cold phosphate buffer saline (PBS) before direct lysis in sample buffer (62.5 mM Tris-HCl (pH 7.6), 10% (*v/v*) glycerol, 2% (*w/v*) SDS, 50 mM fresh dithiothreitol. Samples were sonicated before boiling for 10 min at 95°C. Samples were centrifuged at 17,000 × g for 10 min. Protein concentration was determined at OD660 using Pierce^™^ reagent supplemented with ionic detergent compatibility reagent (Thermo Fisher Scientific, Oxford, UK). Protein was separated by denaturing polyacrylamide gel electrophoresis using gradient Invitrogen NuPage^™^ protein gels (ThermoFisher Scientific, Oxford, UK). Resolved proteins were transferred to Immobilon^®^-P polyvinylidene difluoride membranes (Merck Life Science, Dorset, UK). Western blotting was carried out as directed by the antibody manufacturer’s protocols; primary antibodies (Cell Signaling Technology Danvers, USA) and horse radish peroxidase-conjugated secondary antibodies (Merck Life Science, Dorset, UK). Protein bands were detected by enhanced chemiluminescence using Cytiva Amersham^™^ ECL select^™^ western blotting detection reagent (Cytiva, Buckinghamshire, UK).

### Soft agar colony formation assay

BD DIFCO^™^ Noble Agar (BD BioSciences, Wokingham, Berkshire, UK) was melted in PBS to 1.2% (*w/v*), then diluted in DMEM to yield 0.6% (*w/v*) agar. 2 mL of this solution was added to 6-well plates and was left at room temperature to solidify. In each well, 0.3% (*w/v*) agar DMEM solution containing 20,000 cells was overlaid on top of the 0.6% (*w/v*) agar bottom layer. After setting, media containing the relevant drugs was added and cell colonies were grown between 2–4 weeks, with the media changed every 72 h to refresh drugs. Images were taken on an EVOS XL Core camera and analysed in ImageJ. (v.53) to determine colony diameters. After drug treatment duration, the media was changed and replaced every 72 h in the absence of drugs for a further 3 weeks and further images were taken.

### RNA-sequencing

Cells were washed in ice cold PBS and lysed in RNAprotect^®^ Cell Reagent (Qiagen, West Sussex, UK). RNA was extracted using QIAshredder^®^ and RNAeasy^®^ Mini kits (Qiagen, West Sussex, UK) and were stored at −80°C. RNA library preparation and sequencing were performed through a commercial service/collaboration with Wales Gene Park (Cardiff University, UK), as described previously [24], except the Illumina^®^ TruSeq^®^ RNA sample preparation v2 kit (Illumina Inc, Great Abington, Cambridgeshire, UK) was used for library preparation, according to the manufacturer’s instructions. Following validation, the libraries were normalised to 8 nM and the pool was sequenced on the MiSeq with a 150 cycle, version 3, cartridge (both Illumina Inc) according to the manufacturer’s instructions. Differentially expressed transcripts were identified using the DeSeq2 package in R [25]. Analysis was carried out on all pairwise comparisons in the dataset. P-values were corrected for multiple testing using the Benjamini-Hochberg false discovery rate (FDR) method. Bioinformatic work was initially carried out by Wales Gene Park.

### Patient-derived TSC transcriptomic analysis and gene ontology analysis

Samples of TSC patient-derived tumours (*n* = 15) were collected by Prof. J. MacKeigan (Michigan State University, Grand Rapids, MI, USA). Gene expression analysis was performed as described [26]. Differentially expressed gene (DEG) analysis was performed with GeneAnalytics (LifeMap Sciences Inc., Covina, CA, USA). A similar analysis was performed with TSC patient-derived cortical tubers (*n* = 15). Gene ontology analysis was used to identify dysregulated inflammatory and immune system processes in TSC patient-derived tumours. Datasets were imported into Microsoft Excel to generate volcano plots.

### Transcriptional activation ELISAs

Cells were seeded on 6 cm plates and grown over two days until they reached 80–90% confluency. Media was replaced with serum-depleted media, including pathway inhibitors or DMSO, where applicable, for 24 h. When assaying cytokine induction, media was supplemented with TNFα or IL-6 for the final 2 or 1 h of treatment, respectively. When assaying the effect of media conditioned by *TSC2*-deficient cells on wild-type cells, *TSC2*-deficient MEFs or AML cells were grown until 80% confluency before the media was replaced with serum-free media. Cells remained under starved conditions for 24 h before the conditioned media was collected, briefly centrifuged, and then added to wild type cells to stimulate them. For transcription assays, cells lysates were prepared and were assayed using TransAM^®^ STAT3 Transcription Factor ELISA Kit (Active Motif, Waterloo, Belgium) with nuclear preparations following the manufacturer instructions.

### Conditioned media ELISAs

Secreted IL-6 and VEGF-A concentrations in the media were measured using R&D Systems Duoset ELISAs and ancillary reagent kits (Bio-Techne Ltd., Abingdon, UK) as per the manufacturer instructions. Cells were grown in 12-well plates to 90% confluency. Serum-supplemented media was replaced with serum-supplemented media containing drug treatments. Post-treatment, media was collected, centrifuged (1 min at 13,000 rpm), and stored on ice. Samples were diluted 1:10 and loaded onto plates precoated with capture antibody. Absorbance was measured at OD_450_ using a BioTek Cytation 3 plate reader, with wavelength correction applied at OD_540_.

### Wound scratch cell migration assays

Cells were seeded at a high confluency in 12-well plates (350,000 cells/well) and grown to full confluency overnight. Next, cells were scratched in a straight vertical line using a 200 μL pipette tip to form a wound within the confluent cell layer. Media was next aspirated before being replaced with serum starved media (2% (v/v) FBS) including the drug to be assayed or vehicle (DMSO). “Wounds” were immediately imaged via stereomicroscopy at 4x, and a pen marking was made for later reference of the area to be observed. At 24 and 48 h, wounds were imaged again to visualise closure of the wound over time. The area of wound scratches was calculated in ImageJ and closure was recorded as a percentage.

### Quantitative reverse transcription PCR (qRT-PCR) analysis

*TSC2*(−) or *TSC2*(+) AML cells were grown to 70% confluency. Media was replaced with serum- depleted media for 24 h prior to cell collection in RNAprotect (Qiagen, West Sussex, UK) and then stored at − 80°C. RNA was isolated using the RNeasy Plus Mini Kit (Qiagen, West Sussex, UK) and cDNA was generated with the Reverse Transcriptase Core Kit (Eurogentec, Belgium). qRT-PCR was performed using TakyonTM ROX Sybr MasterMix dTTP blue (Eurogentec, Belgium). Ct values were normalised to IPO8 and TUBA1A. Primers were purchased from Integrated DNA Technologies and optimised for annealing temperature and efficiency. PDCD1LG2 forward primer GAACCCAGGACCCATCCAAC and reverse primer TTCAGATAGCACTGTTCACTTCCC and 183 bp amplicon length; IPO8 forward primer ACTGTTGCACATTGTTAGAG and reverse primer ACTTTGCCAAATATCTCAGC and 138 bp amplicon length; TUBA1A forward primer TCTTCCACCCTGAGCAACTT and reverse primer GGAAAACCAAGAAGCCCTGG and 159 bp amplicon length. Dissociation curves were carried out to verify specificity of primer sets.

### Statistical analysis

Protein band intensities were quantified using ImageJ. (v.53). Band intensity was normalised to β-actin expression. Fold changes were normalised to the DMSO control, where applicable. Normalised data were inputted into GraphPad Prism9 (Dotmatics, Boston MA USA) and statistical analysis was carried out. Normality testing in Prism9 was carried out with a D’agostino & Pearson and Shapiro-Wilk test. Normally (Gaussian) distributed data was then analysed by an ordinary one-way ANOVA with Tukey’s multiple comparisons or two-way ANOVA with Šídák’s multiple comparisons. When analysing 2 groups only, a parametric unpaired t-test was carried out. Data are presented as mean ± SEM. Non-normally distributed data were assessed by the Kruskal-Wallis test, with Dunn’s multiple comparisons tests. If the comparison was between only two groups, nonparametric Wilcoxon t-tests were instead carried out. P-values: * < 0.05, ** < 0.01, *** < 0.001, **** (or #)

## Results

### TSC2 loss is characterised by dysregulated expression of NF-κB genes

**Figure 1:**
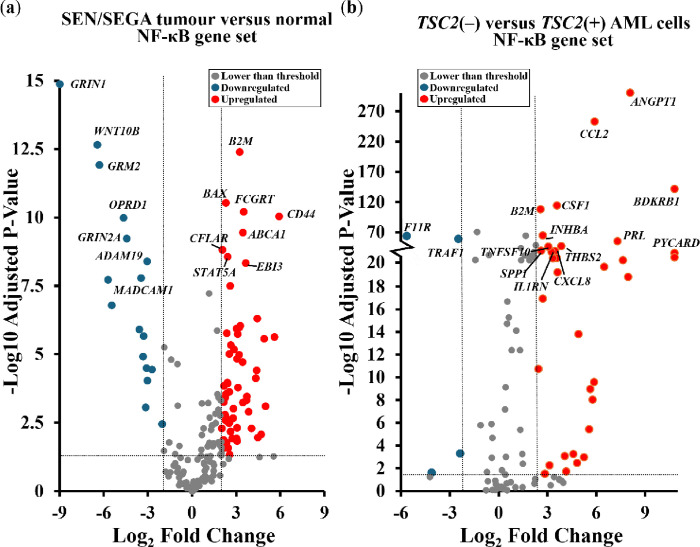
NF-κB regulatory and target genes are differentially expressed in TSC. Volcano plots show differential expression of NF-κB-linked genes from (**a**) TSC patient-derived brain tumours (*n* = 15), and (**b**) cultured *TSC2*(−) AML lacking functional TSC2 (*n* = 6), when compared to non-TSC brain tissue and *TSC2*-rescued *TSC2*(+) AML cells, respectively. The 15 most significantly dysregulated genes are labelled. Horizontal dashed lines represent adjusted p-value = 0.05; vertical dashed lines represent a Log_2_ fold change of ±2. Sample collection, analysis, and statistics for the dataset used in volcano plots were performed by Martin *et al*. [26].

To explore dysregulated gene expression in TSC, mRNA sequencing (RNAseq) data from 20 TSC patient SEN/SEGAs was compared to non-TSC brain tissue (as previously described [26]), and also RNAseq from *TSC2*(−) AML cells (621 – 101) was compared to *TSC2*(+) AML cells (621 – 103). Gene ontology analysis of differentially expressed genes indicated enrichment of inflammatory and immune response genes within TSC patient-derived tumours (supplementary data), as previously described [26]. To better understand these dysregulated inflammatory pathways in TSC, we analyzed expression of 190 regulatory and NF-κB target genes. This NF-κB-linked gene set was adapted from a list developed by the Gilmore lab (Boston University) [27]. Volcano plots of differentially expressed genes illustrate dysregulation of NF-κB-linked genes in TSC patient-derived brain tumours (Fig. 1a), and *TSC2*(−) AML cells (Fig. 1b) when compared with their respective wild-type controls.

The observed transcriptional signature suggests a redox imbalance that could create a tumour microenvironment of oxidative stress and inflammation. Within both *in vivo* and *in vitro* datasets, NF-κB-related genes were significantly dysregulated. Within SEN/SEGAs, a total of 47 significantly upregulated NF-κB regulatory and target genes was observed (over Log2 fold change of 2 and adjusted p-value < 0.05), compared to 19 significantly downregulated genes (below Log2 fold change – 2 and adjusted p-value < 0.05). This pattern of NF-κB dysregulation persisted within cortical tubers (17 NF-κB-linked genes increased and 2 decreased; supplementary data, Fig. 1) and *TSC2*(−) AML cells (34 NF-κB-linked genes increased and 4 decreased). As we saw a greater abundance of upregulated NF-κB linked genes, we hypothesized that the NF-κB pathway was activated in TSC. To follow on from this, we next assessed the activity of the NF-κB pathway within *in vitro* TSC cell line models.

### Altered pathway regulation of NF-κB and STAT3 in TSC2-deficient cells

**Figure 2:**
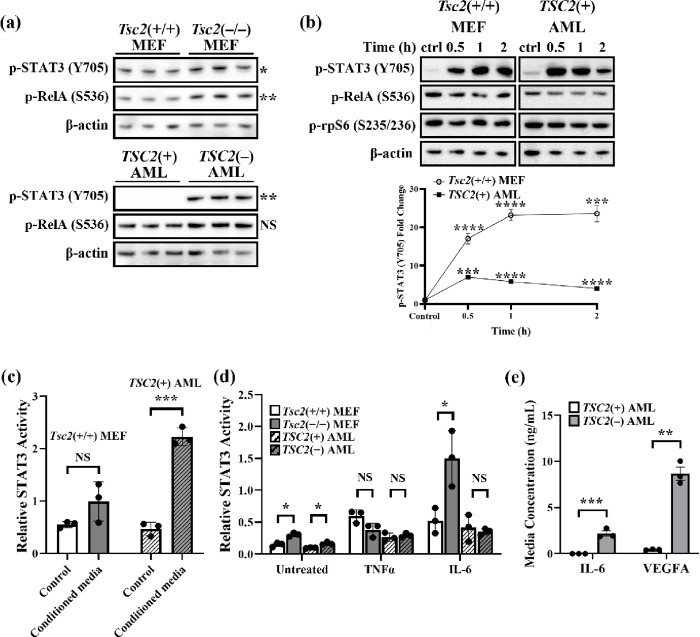
Complex NF-κB and STAT3 signalling interplay in TSC. (**a**) Confluent cells were serum-starved for 24 h and lysed. Western blot analysis of S536-phospho RelA and Y705-phospho STAT3 was carried out in *Tsc2*(−/−)MEF (top panel) and *TSC2*(−) AML cells (bottom panel), respectively. b-actin was used as a loading control (western blot panel shows n = 3, unpaired t test). (**b**) Serum-starved *Tsc2*(+/+) MEF or *TSC2*(+) AML cells were stimulated with conditioned media from serum-starved *Tsc2*(−/−) MEF or *TSC2*(−) AML cells, respectively, and western blot analysis of S536-phospho RelA, Y705-phospho STAT3, S235/236-phospho rpS6 and b-actin as a loading control was assayed at 0.5, 1 and 2 h of treatment duration. Densitometry analysis of Y705-phospho STAT3 is also shown (*n* = 3, one-way ANOVA). (**c**) The control and 1 h treatment condition from (b) were subjected to STAT3 transcription assays (*n* = 3, unpaired t test). (**d**) Serum-starved *Tsc2*(−/−) and *Tsc2*(+/+) MEFs, and *TSC2*(−)and *TSC2*(+) AML cells were stimulated with either 30 ng/mL TNFα for 2 h or 50 ng/mL IL-6 for 1 h, as indicated. STAT3 activity assays were carried out (*n* = 3, unpaired t-tests). (e) The media concentration of IL-6 and VEGFA was compared between *TSC2*(+) and *TSC2*(−) AML cells by ELISA (*n* = *3*, unpaired t-test).

STAT3 is a downstream target of NF-κB, and these two pathways are closely linked [11]. Prior research indicates that STAT3 signalling is enhanced in *TSC2*-deficient cells [19, 28]. We sought to characterise the activity of NF-κB and STAT3, including cytokine responsiveness, in TSC cell models. For this, we used *TSC2*(−) or *TSC2*(+) AML cells as well as *Tsc2*(+/+) or *Tsc2*(−/−) murine embryonic fibroblasts (MEFs). We observed increased phosphorylation of S536-RelA and Y705-STAT3 in both *Tsc2*(−/−) MEF and *TSC2*(−) AML cells, compared to their respective TSC2-expressing controls (Fig. 2a). As these phosphorylation sites are required for activity of RelA and STAT3, this data implies that both NF-κB and STAT3 become more transcriptionally active upon loss of *TSC2*. To explore potential autocrine signalling crosstalk to STAT3, the wild-type control cells were stimulated with conditioned media that was taken from their respective untreated serum-starved *TSC2*-deficient cell line (Fig. 2b), and STAT3/NF-κB pathway activation was assayed by western blot. Supplementation of conditioned media (obtained from *TSC2*-deficient cells) caused acute STAT3 activation within both wild-type cell lines, suggesting that *TSC2*-deficient cells secrete factors that potently induce the STAT3 pathway. This was confirmed by STAT3 transcriptional activation ELISA, wherein the wild type *Tsc2*(+/+) MEF and *TSC2*(+) AML cells were treated with their matched *TSC2*-deficient cell conditioned media for 1 h, causing a large upregulation in STAT3 nuclear activation (Fig. 2c). Next, we tested whether TSC2 expression affected the transcriptional activity of STAT3 induced by cytokines, using 2 h TNFα (30 ng/mL) or 1 h IL-6 (50 ng/mL). While STAT3 activation after TNFα and IL-6 was similar in the *TSC2*(−) and *TSC2*(+) AML cells, *Tsc2*(−/−) MEFs had higher sensitivity to IL-6 treatment, where a 4.9-fold STAT3 induction was observed (Fig. 2d). Conversely, *Tsc2*(+/+) MEFs demonstrated a 3.5-fold increase in STAT3 activity following IL-6 stimulation. Within STAT3 transcription ELISAs, *Tsc2*(−/−) MEFs appeared to have a less significant response to TNFα, when compared to *Tsc2*(+/+) MEFs. Based on these data, we hypothesised that the *TSC2*(−) AML cells release more cytokines, which in turn enhances inflammatory autocrine signalling. Using ELISA, we confirmed a > 19-fold increase in VEGF-A in conditioned media taken from *TSC2*(−) AML cells (Fig. 2e. IL-6 secretion was not detected in *TSC2*(+) AML cells but was significantly increased in *TSC2*(−) AML cells.

### NF-κB inhibition reduces STAT3 activation

**Figure 3:**
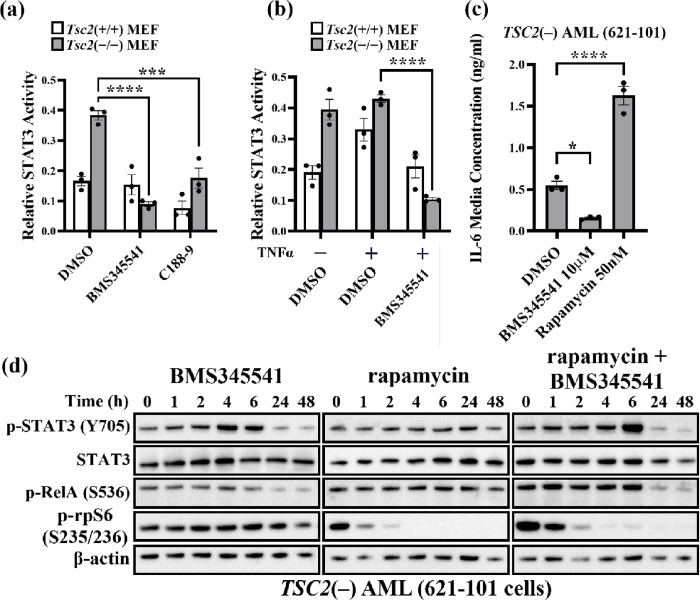
Autocrine signalling crosstalk between NF-κB and STAT3 in TSC cell models. STAT3 activity assays were carried out from nuclear lysates prepared from *Tsc2*(−/−) and (+/+) MEFs treated with either (**a**) DMSO control, 5 μM BMS345541 or 15 μM C188–9 for 24 h or (**b**) TNFα (30 ng/mL, 2 h treatment) after pre-treatment with 5 μM BMS345541 for 24 h, as indicated (*n* = *3*, two-way ANOVA with Tukey’s multiple comparisons). (**c**) *TSC2*(−) AML cells were treated with either DMSO control, 10 μM BMS345541, or 50 nM rapamycin for 24 h. Conditioned media from these cells were subjected to IL-6 ELISAs (*n = 3*, one-way ANOVA with Tukey’s multiple comparisons). (**d**) Phosphorylation status of RelA, STAT3, and rpS6 were investigated following a time-course (0, 1, 2, 4, 6, 24, and 48 h) with either 5 μM BMS345541 or 50 nM rapamycin, as single or combination treatments (*n= 3*).

Next, we examined whether NF-κB inhibition could diminish the heightened activity of STAT3 in *TSC2*-deficient cells. To do this, *Tsc2*(−/−) MEFs and *Tsc2*(+/+) MEFs were treated with 5 μM BMS345541 (an IKK complex allosteric inhibitor), and the transcriptional activity of STAT3 was measured. In *Tsc2*(−/−) MEFs, STAT3 activity was reduced after 24 h of BMS345541 treatment, but not in the *Tsc2*(+/+) MEFs (Fig. 3a). As a control, we used C188–9, a STAT3 inhibitor, in both the *Tsc2*(−/−) and *Tsc2*(+/+) MEFs. C188–9 reduced STAT3 activity in both *Tsc2*(+/+) and *Tsc2*(−/−) MEFs. C188–9 and BMS345541 both reduced STAT3 activity to a similar level, demonstrating that heightened NF-κB activity in *Tsc2*(−/−) MEFs may be responsible for the observed upregulation in STAT3. NF-κB inhibition with BMS345541 also blocked TNFα induced activation of STAT3 in *Tsc2*(+/+) MEFs (Fig. 3b), showing that NF-κB is required for cytokine-induced STAT3 induction. Since we previously demonstrated that *TSC2*-deficient cells secrete high levels of IL-6, it is likely that STAT3 activity and IL-6 secretion are linked. Furthermore, IL-6 was recently shown to be over-expressed in TSC2-disease models, and inhibition with IL-6 antibody antagonists was shown to reduce tumour growth [29]. Therefore, we next aimed to investigate whether NF-κB inhibition could regulate IL-6 secretion. *TSC2*(−) AML cells were treated with 10 μM BMS345541 or 50 nM rapamycin for 24 h, and IL-6 levels were measured by ELISA. BMS345541 reduced IL-6 secretion by approximately 4-fold, whereas rapamycin increased IL-6 secretion by approximately 3-fold (Fig. 3c). Next, we investigated how NF-κB inhibition may reduce STAT3 phosphorylation on Tyr705 over a 48 h period of treatment, when compared to either rapamycin or a combination of both drugs. Rapamycin was used to determine the effects of mTORC1 inhibition on NF-κB and STAT3 activity. We also aimed to observe if the impact of NF-κB inhibition on STAT3 activity was mTORC1-dependent. Surprisingly, we identified a biphasic response to NF-κB inhibition in *TSC2*(−) AML cells, with an initial increase in Y705-STAT3 phosphorylation that then dropped at the 24 and 48 h time points (0.8-fold and 0.6-fold, respectively) (Fig. 3c). Rapamycin showed little effect on RelA or STAT3 phosphorylation but did ablate rpS6 phosphorylation, as expected. Meanwhile, a combinatorial treatment of BMS345541 and rapamycin dampened the increase in STAT3 phosphorylation at 6 h (3.2-fold increase for BMS345541 versus 1.58-fold increase for combinatorial treatment). Combinatorial treatment of BMS345541 and rapamycin also reduced the total levels of STAT3 at later timepoints, whereas BMS345541 treatment did not elicit this effect. At the later time points of 24 and 48 h, combinatorial treatment of BMS345541 and rapamycin was more potent at reducing STAT3 phosphorylation (supplementary data, Fig. 2).

### NF-κB inhibition reduces anchorage-independent growth and cell migration in TSC2-deficient cells

**Figure 4:**
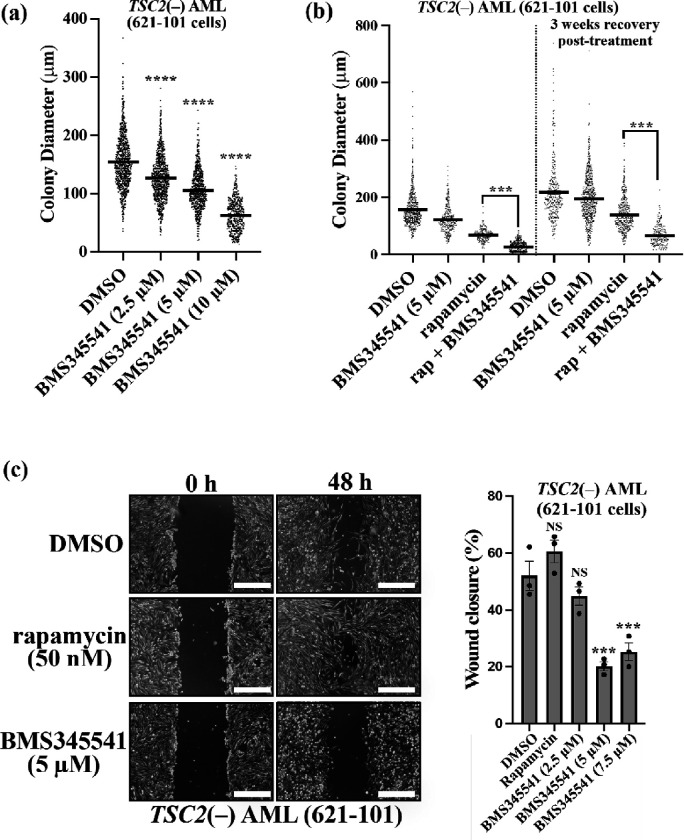
NF-κB inhibition reduces anchorage-independent growth and migration in *TSC2*-deficient cells. (**a**) *TSC2*(−) AML cells were grown over 3 weeks in soft agar, supplemented with increasing concentrations of BMS345541 (0, 2.5, 5, and 10 μM), where indicated. 30 phase contrast images were taken per condition and diameter of all visible colonies were determined in ImageJ. (v.53) [32]. (Kruskal-Wallis test with Dunn’s multiple comparison tests.) (**b**) Same as for panel ‘a’ with the inclusion of 50 nM rapamycin as a single or combinatory treatment with BMS345541. Colony diameter was recorded before media was replaced with untreated media for a further 3 weeks in the absence of drug. (**c**) Wound scratch assays were carried out on *TSC2*(−) AML cells in the presence of DMSO vehicle only control, rapamycin (50 nM) and BMS345541 (5 μM). The ‘wound’ was imaged after 48 h and wound closure (%) was calculated using ImageJ. (v.53). Representative images are shown, 500 μm scale bar (two-way ANOVA with Tukey’s multiple comparison tests, *n* = 3).

To explore whether NF-κB inhibition might limit tumorigenesis, *in vitro* colony growth assays were carried out. Colonies of *TSC2*(−) AML cells were grown over 3 weeks in soft agar with increasing doses of BMS345541. BMS345541 at 10 mM was the most effective drug concentration, reducing anchorage-independent growth nearly 3-fold (Fig. 4a). Additionally, 10 μM BMS345541 treatment reduced the number of colonies by half, when compared to DMSO (989 versus 538 colonies). As TSC patient tumours regrow after discontinuation of mTORC1 inhibitors [10], rapamycin was also compared as a single drug treatment and in combination with BMS345541 (Fig. 4b). Anchorage-independent growth was assessed after 3-weeks of drug treatment. Overall, reduced colony growth was observed in the presence of BMS345541, and a combinatory treatment of BMS345541 and rapamycin showed a more potent effect. To explore drug recovery, anchorage-independent growth was further evaluated after removal of the drug for a further 3 weeks. Importantly, combined treatment with BMS345541 and rapamycin markedly reduced anchorage-independent growth upon discontinuation of treatment, which was more effective than treatment with rapamycin alone. Anchorage-independent growth assays were also performed with both MEF and ELT3 TSC cell models that showed a similar trend of colony growth reduction with NF-κB inhibitor (supplementary data, Fig. 3).

Lastly, we investigated the effects of NF-κB inhibition on the cell migration of *TSC2*(−) AML cells. NF-κB is known to influence migration and metastasis in cancers [30], and migration is also a key feature of lymphangioleiomyomatosis (LAM) that can occur in TSC [31]. To do this, wound scratch assays were carried out in reduced-serum media containing BMS345541 over two days. We observed a reduction in migration within cells treated with BMS345541, whereas rapamycin was ineffective at reducing migration (Fig. 4f).

### 2.5. The immune checkpoint protein PD-L2 is dysregulated in TSC via inflammatory signalling

**Figure 5:**
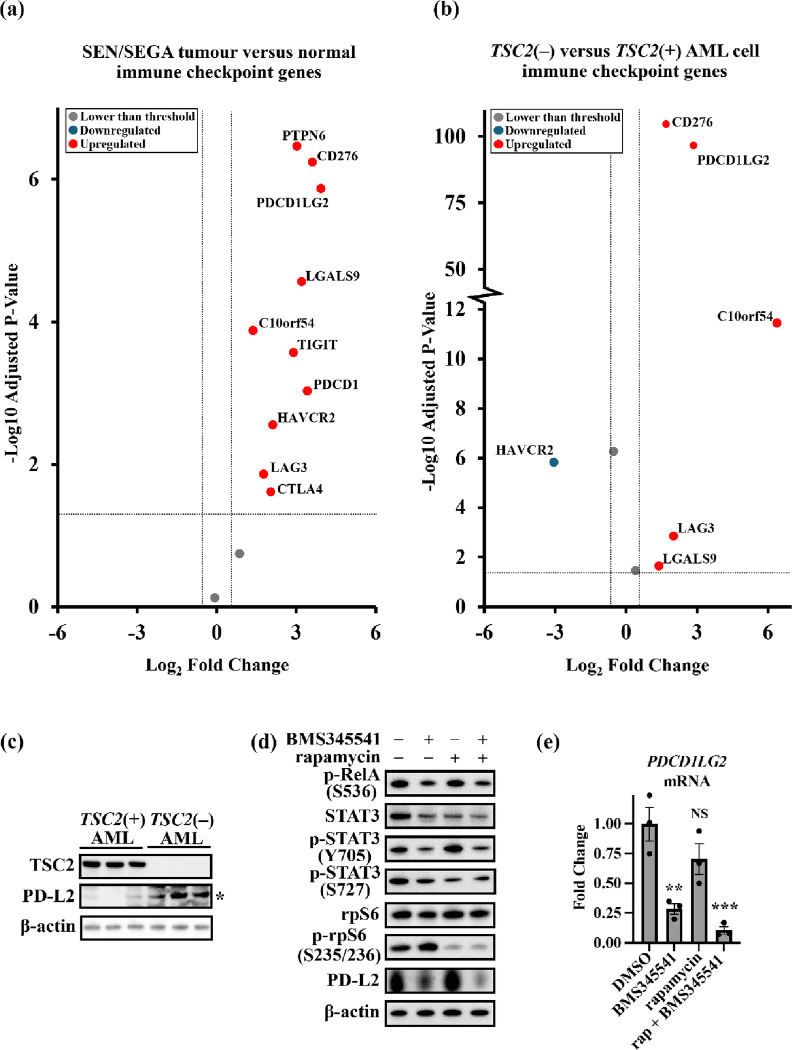
Immune checkpoint genes are dysregulated in TSC. Volcano plots show differential expression of immune checkpoint genes from (**a**) TSC patient-derived brain tumours (*n* = 15), and (**b**) cultured *TSC2*(−) AML lacking functional TSC2 (*n* = 6), when compared to non-TSC brain tissue and *TSC2*-rescued *TSC2*(+) AML cells, respectively. All significantly dysregulated genes are labelled. Horizontal dashed lines represent adjusted p-value = 0.05; vertical dashed lines represent a Log_2_ fold change of ±2. Sample collection, analysis, and statistics for the dataset used in volcano plots were performed by Martin *et al*. [26]. (**c**) PD-L2 expression was compared between *TSC2*(−) and *TSC2*(+) AML cells (western blot panel shows *n* = 3, unpaired t test). (**d**) Serum-starved *TSC2*(−) AML cells were treated with either vehicle only DMSO, BMS345541 (5 mM), rapamycin (50 nM), or a combination of both drugs for 24 h and proteins phosphorylation and expression was assessed. Densitometry analysis was performed with normalisation to β-actin (*n* = 3, one-way ANOVA with Dunn’s multiple comparison tests). (**e**) Serum-starved *TSC2*(−) AML cells were treated with either vehicle only DMSO, BMS345541 (5 mM), rapamycin (50 nM), or a combination of both drugs for 24 h, and *PDCD1LG2* expression was analysed by RT-qPCR. Expression was normalised to *IPO8* and *TUBA1A* (*n* = 3, one-way ANOVA with Dunn’s multiple comparisons).

Inflammatory signalling from NF-κB can influence leukocyte recruitment and modulation, which is a disease facet that has been reported in TSC patient tumours [26]. Given these connections in TSC and immune signalling, we next compared the differential expression of immune checkpoint genes in both SEN/SEGA (Fig. 5a) and *TSC2*(−) AML cells (Fig. 5b). This set of immune checkpoint regulators was adapted from a list on ACROBiosystems [33]. Of note, we observed heightened expression of *PDCD1LG2*, which is a negative regulator of T-cells that can be expressed on stromal and/or tumour cells to repress immune recognition [34]. PD-L2 protein expression was markedly enhanced in *TSC2*(−) AML cells when compared to the wild-type control (Fig. 5c) and its expression was ablated when NF-κB was inhibited with 5 μM BMS345541 (Fig. 5d). Inhibition of mTORC1 with rapamycin was unable to reduce the high protein expression of PD-L2 in these TSC-disease cells. Similarly, gene expression of *PDCD1LG2* was reduced after treatment with 5 μM BMS345541, but not after inhibition of mTORC1 with rapamycin (Fig. 5e).

## Discussions

The NF-κB pathway plays a key role in the progression of many cancers and inflammatory conditions via the upregulation of pro-inflammatory genes. While inflammation is a known feature of TSC-linked tumours, the role that NF-κB plays in the disease pathology of TSC is poorly understood. This study aimed to elucidate the status of NF-κB in TSC, and thus identify the potential role that NF-κB signalling has in TSC pathogenesis. Through our findings, we show that NF-κB becomes dysregulated in TSC patient tumours and cell line models. Our data implies that mTORC1 inhibitor therapies are unlikely to restore inflammation in TSC, raising the possibility that NF-κB dysregulation could contribute to the failure of current mTORC1 inhibitors to completely ablate TSC symptoms [10].

This study highlights that dysregulated NF-κB and STAT3 signalling contributes to the observed inflammatory signature found within TSC cell line models and in TSC patient tumours. Such inflammatory signals are likely linked to TSC-associated symptoms. For instance, neuroinflammation is linked to a variety of neuropsychiatric conditions, including TSC-associated neuropsychiatric disorders (TANDs) and neurodegenerative disorders. Neuroinflammation has also been characterised in schizophrenia and depression [35, 36]. A review by Matta *et al*. highlights the prevalence of neuroinflammation within autism spectrum disorder [37], while a review by Aronica and Crino categorises the dominant role of neuroinflammation in epilepsy [38]. As hyperactivation of STAT3 is a known driver of epilepsy [39, 40], STAT3 (and NF-κB) might be connected to the neurological symptoms associated with TSC. Cortical tubers are a suspected focal point of epilepsy in TSC. Inflammation through NF-κB activity may contribute to epileptogenic signalling. This is supported by enhanced NF-κB dysregulation in cortical tubers (supplementary data, Fig. 1).

NF-κB and STAT3 are closely linked with multiple mechanisms of signalling cross talk (reviewed in [11]). The complex signalling interplay between NF-κB and STAT3 is evident and may partially explain the observed variation in the state of NF-κB activity in related TSC research studies [18]. Many cytokines are NF-κB responsive and these include IL-6 [41]. Consequently, NF-κB can indirectly activate STAT3 via a positive feedback loop, where IL-6 secretion will induce STAT3 activation via interleukin receptors. Signalling crosstalk between the NF-κB and STAT3 was apparent in cell line models of TSC, which is a feature shared in cancer [42], including glioma [43]. Potentially, inhibition of one component in this feedback loop may be sufficient to dampen down this inflammatory signal. Arguably, mTORC1 activation has been shown to contribute to NF-κB signalling, so standard therapy with mTORC1 inhibitors in TSC should have some capacity to dampen down the inappropriate activity of NF-κB [44]. In our cell line models, we showed that secreted cytokines such as IL-6 likely contribute to STAT3 activation in TSC. NF-κB inhibition could reduce STAT3 activity in *TSC2*-deficient cells, and this was likely through inhibition of IL-6 signalling. Rapamycin was ineffective at reducing IL-6 secretion and STAT3 activity in *TSC2*-deficient cell lines. Following on from this, combinatorial treatment of mTORC1 inhibition and NF-κB inhibition Page 12/23 was sufficient to reduce STAT3/NF-κB and mTORC1 signalling. However, it is important to note that in the cell line studies presenting here, treatment with mTORC1 inhibitors were only carried out over short time periods (up to 3 days). It is possible that longer duration of mTORC1 inhibition would be required to reduce chronic inflammation in TSC-associated tumours and/or neuroinflammation. Supporting this line of thought, Everolimus (a rapalogue) shows greater efficacy in TSC patients to reduce seizures after longer durations of treatment, i.e., up to 3 years of treatment [9].

Rapamycin is a cytostatic drug that has potency to stabilise disease in TSC. Through anchorage-independent growth assays we demonstrate the cytostatic drug property of rapamycin. While rapamycin causes marked reduction in growth, cell colonies quickly recover and grow after the end of rapamycin treatment. While single drug inhibition of NF-κB showed little long-term effectiveness to repress anchorage-independent growth of *TSC2*(−) AML cells, we observed marked reduction of colony size with combined treatment with NF-κB/mTOR inhibitors, which persisted after removal of both drugs.

Lastly, we aimed to identify dysregulated targets which were insensitive to mTORC1 inhibition. A high degree of immune cell infiltration likely contributes to the disease pathology of TSC, however TSC-derived tumours appear to avoid being attacked by the immune system. This is likely due to upregulated immune checkpoint regulators that can be presented on stromal and/or tumour cells, such as PD-L2. Other studies have identified that STAT3 signalling can upregulate PD-L2 [45, 46]. In this study, we identified that STAT3 activity was linked to NF-κB activity in *TSC2*-deficient cells. Our findings indicate that PD-L2 could be downregulated with NF-κB inhibition in *TSC2*-deficient cells that could be due to inhibition of STAT3. Our data show that combinatorial inhibition of NF-κB and mTORC1 is effective for inhibiting both mTORC1 sensitive and insensitive targets. Further investigation is necessary to identify whether other immune checkpoint regulators may also be regulated through dysregulated inflammatory signalling in TSC. This work implies that combination therapy to target both NF-κB and mTORC1 might have longer lasting benefits to treat tumours in TSC.

## Conclusions

NF-κB signaling is dysregulated and likely contributes to inflammation/immune signalling in TSC. Facets of this dysregulated inflammatory/immune signalling are not directly regulated by mTORC1 but may be restored via NF-κB pathway inhibitors. Therefore, the NF-κB signalling pathway presents itself as a possible therapeutic target for the treatment of TSC, and combinatory approaches with traditional mTORC1 inhibitors may prove more effective as an adjunct therapy.

## Data Availability

Raw data for RNAsequencing of TSC patient tumours was previously deposited in the Database of Genotypes and Phenotypes (dbGaP) under the accession code phs001357.v1.p1 [26]. All datasets generated or analyzed during this study are either included in this article or supplementary files. The data analyzed during the current study are available from the corresponding author upon reasonable request.
